# Special Issue “Advanced Composites: From Materials Characterization to Structural Application”

**DOI:** 10.3390/ma13245820

**Published:** 2020-12-21

**Authors:** Viktor Gribniak

**Affiliations:** 1 Department of Steel and Composite Structures, Vilnius Gediminas Technical University (Vilnius Tech), Saulėtekio av. 11, LT-10223 Vilnius, Lithuania; Viktor.Gribniak@vgtu.lt; Tel.: +370-6134-6759; 2 Laboratory of Innovative Building Structures, Vilnius Tech, Saulėtekio av. 11, LT-10223 Vilnius, Lithuania

The modern industry allows synthesizing and manufacturing composite materials with a wide range of mechanical properties applicable in medicine, aviation, automotive industry, etc. Construction production generates a substantial part of budgets worldwide and utilizes vast amounts of materials. Nowadays, the practice has revealed that structural applications of innovative engineering technologies require new design concepts related to the development of materials with mechanical properties tailored for construction purposes. It is the opposite way to the current design practice where design solutions are associated with the application of existing materials, the physical characteristics of which, in general, are imperfectly suiting the application requirements.

The efficient utilization of engineering materials is the result of achieving the solution of the above problem. The efficiency can be understood in a simplified and heuristic manner as the optimization of the performance and the proper combination of structural components, leading to the utilization of the minimum volume of materials; consumption of the least amount of natural resources should ensure the development of more durable and sustainable structures. The solution of the eco-optimization problem, based on the adequate characterization of the materials, enables implementing principles of environment-friendly engineering when the efficient utilization of advanced materials guarantees the structural safety required.

The identification of fundamental relationships between the structure of advanced composites and the related physical properties was the focus of the completed Special Issue. The research team from the Democritus University of Thrace achieved outstanding results in the development and analysis of fibrous reinforcement, improving the mechanical performance of structural components [1,2,3]. The manuscripts [1,2] present new experimental results of cyclic tests of fiber-reinforced concrete beams with bar reinforcement. The reported outcomes are a valuable reference for further analysis and development of advanced cement-based composites. The application of the steel fibers improved the cracking resistance and failure ductility of concrete beams. However, the success of this solution requires a suitable proportioning of the concrete. An extensive review of the literature is a beneficial attribute of the article [2]. The listed publications adequately describe the current state of the art and determine the unsolved problems for further research. This paper contributes to the limited existing literature on cyclic tests of reinforced concrete beams with steel fibers, providing detailed experimental data of beams with a relatively high amount of steel fibers (3%) that has not been examined in structural applications. The work [3] develops a numerical (finite element) model, investigating short-term deformation behavior of structural concrete elements reinforced with a combination of steel fibers and bar reinforcement. The comparison of the numerical and experimental results reveals reliability and computational-efficiency of the developed model, capturing well the critical aspects of the mechanical response of fiber-reinforced cement-based composites and the favorable contribution of the steel fibers on the residual stiffness and ductility of such structures.

The articles [4,5] expand the application of fiber-reinforced polymer (FRP) reinforcement to the strengthening of concrete structures. The paper [4] investigates the local bond stress–slip effects on the global response of the near-surface-mounted (NSM) FRP-strengthening systems in terms of load-bearing capacity, bonding mechanisms, slip resistance, and distribution of shear stresses and strains along the bonded length. A numerical procedure suitable for the design of strengthening systems was developed as well. The calculations employ the finite difference method to solve the governing equations describing the mechanical resistance of the FRP-to-concrete joint. The model was verified experimentally using pull-out shear tests of FRP strips. The article [5] investigates material behavior within the multiple-component system of a structural concrete element strengthened in flexure with an externally bonded fiber-reinforced polymer plate. The specific feature of the proposed approach is the possibility to track the evolution of the damage accumulation in concrete, which is related directly to the initiation of micro-cracks and further propagation of cracks at the macroscopic scale.

The articles included in the Special Issue explore the development of sustainable composites with valorized manufacturability for a breakthrough from conventional practices and corresponding to the Industrial Revolution 4.0 ideology. The publications revealed that the application of nano-particles improves the mechanical performance of composite materials [6]; advanced woven fabrics efficiently reinforce soft body-armor [7]; heat-resistant aluminum composites ensure the safety of overhead power transmission lines [8]; chemical additives can help in detecting temperature impact on concrete structures [9]. The publications [10,11] investigated connection problems of high-performance composites. The sound radiation of shape memory alloy (SMA) composite laminates is the focus of interest of the paper [12]. The study demonstrates that composite laminates embedded with SMA wires have an excellent ability to alter sound radiation characteristics and adjusting modal frequencies. That provides a solid rationale for diminishing the resonance problems and reducing the vibrations and sound radiation of composite laminates from the point of the view of materials engineering. The article [13] analyzes tribological properties and resonance problems of polymeric composites utilized in the aviation industry. The research confirmed several hypotheses related to the abrasive wear process of new materials.

The publication [14] investigates the application of lightweight aluminum honeycombs for developing an efficient system for blast protection. The review article [15] is a valuable reference of design solutions of acoustic coatings. Based on the requirements of underwater acoustic stealth, the classification and research background of acoustic coatings are discussed.

The application of advanced manufacturing technologies is the research focus of articles [16,17]. Supersaturated Cu-Cr solid solution was formed in the reference [16] using the mechanical alloying method. Extension of the high-energy milling procedure presents new insights in the field of research where many articles have been published in the past ten years. A prefabricated Ti-based alloy plate was processed in the study [17] using laser surface remelting technology to obtain an in situ, strongly binding amorphous coating. Dental implants and orthopedic prostheses are the common application objects of such alloys because of high corrosion resistance and good biocompatibility.

The articles [18,19,20] investigate problems related to the development of advanced steel structures. It was shown that the refinement of grain size improves the low-temperature toughness of high-strength pipeline steel [18]. The study [19] focuses on the evaluation and analysis of the fracture mechanisms (fracture toughness and microstructural and cracking behavior) of T2 copper-45 steel. The reported results provide a basis for improvement in the performance of welded joints. Although the current trends in the construction industry are related to the use of high-strength steel, the increase in the load-bearing capacity of the member is often not proportional to the rise of the strength of the material. The study [20] employed flexural test results of two groups of structural tubular profiles produced from high- and normal-strength steel to illustrate the above observation. The tests demonstrated that the strength condition controlled the load-bearing capacity of the normal-strength specimens. On the contrary, the stiffness condition governed the failure behavior of the high-strength samples. Thus, identification of the optimum configuration of the cross-section should be the subject for further research.

The published works demonstrate that the choice of construction materials has considerable room for improvement from a scientific viewpoint, following heuristic approaches. At the closure of the Special Issue, altogether, the manuscripts included in the Special Issue were cited 51 times. That highlights the essential impact of the reported outcomes on the research community and their valuable contributions to materials science. The onward Special Issue “Advanced Composites: From Materials Characterization to Structural Application (Second Volume)” of *Materials* continues the successful publication series, aiming to combine the innovative achievements of experts in the fields of materials and structural engineering to raise the scientific and practical value of the gathered results of the interdisciplinary research.
